# *Trichuris suis* ova in relapsing-remitting multiple sclerosis and clinically isolated syndrome (TRIOMS): study protocol for a randomized controlled trial

**DOI:** 10.1186/1745-6215-14-112

**Published:** 2013-04-25

**Authors:** Berit Rosche, Klaus-Dieter Wernecke, Stephanie Ohlraun, Jan-Markus Dörr, Friedemann Paul

**Affiliations:** 1 Department of Neurology and Experimental Neurology, Charité - Universitätsmedizin Berlin, Charitéplatz 1, Berlin, 10117, Germany; 2 Clinical and Experimental Multiple Sclerosis Research Center, Charité - Universitätsmedizin Berlin, Berlin, Germany; 3 SOSTANA GmbH and Charité, Universitätsmedizin Berlin, Berlin, Germany; 4 NeuroCure Clinical Research Center, Charité - Universitätsmedizin Berlin, Berlin, Germany; 5 Experimental and Clinical Research Center, Max Delbrueck Center for Molecular Medicine and Charité - Universitätsmedizin Berlin, Berlin, Germany

**Keywords:** Multiple sclerosis, *Trichuris suis* ova, Clinical trial, Intervention, Immmunomodulation, Hygiene hypothesis

## Abstract

**Background:**

*Trichuris suis* ova is a probiotic treatment based on the hygiene hypothesis. It has been demonstrated as safe and effective in autoimmune inflammatory bowel diseases and clinical trials indicate that helminth infections also have an immunomodulatory effect in multiple sclerosis.

We hypothesize that administering 2,500 *Trichuris suis* ova eggs orally every two weeks for 12 months is - due to its immunomodulatory and anti-inflammatory effect - significantly more effective than oral placebo in preventing new T2 and Gd+ lesions, as quantified by cerebral MRI and clinical examination, in relapsing-remitting multiple sclerosis and clinically isolated syndrome.

**Methods/Design:**

Fifty patients with relapsing-remitting multiple sclerosis or clinically isolated syndrome with clinical activity, not undergoing any standard therapies, will be randomized 1:1 to *Trichuris suis* ova 2,500 eggs every two weeks or matching placebo. The safety, tolerability and effect on disease activity and *in vivo* mechanisms of action of *Trichuris suis* ova in MS will be assessed by neurological, laboratory and immunological exams and magnetic resonance imaging throughout the 12-month treatment period and over a follow-up period of 6 months. Various immunological analyses will be used to assess the overall patient immune response prior to and at varying time points following treatment with *Trichuris suis* ova.

**Discussion:**

We anticipate that *Trichuris suis* ova will be well tolerated and more effective than the placebo in preventing new T2 and Gd+ lesions, as quantified by MRI. We also expect the Th1/Th17 proinflammatory response to shift towards the more anti-inflammatory Th2 response. This study has important clinical implications and will involve extensive research on the immunology of helminth therapy.

**Trial registration:**

ClinicalTrials.gov: NCT01413243

## Background

Multiple sclerosis (MS) is a chronic inflammatory disease of the central nervous system (CNS), whose incidence has increased over recent decades [1] and which features a specific geographical distribution with higher incidence rates in developed Western nations [2,3]. Although the etiology remains unclear, MS is widely assumed to be an autoimmune disease, in which both genetic susceptibility and environmental factors play a role [4]. In 1966, Leibowitz *et al.* first suggested that MS might be associated with high sanitation standards during childhood [5], and subsequent epidemiological surveys have confirmed high MS prevalence in regions where such standards are the norm [6]. Beneficial immunomodulation by helminths in humans has been demonstrated in an observational study of relapsing-remitting (RR) MS patients with asymptomatic, community-acquired gastrointestinal infections [7]. Compared with uninfected MS patients, parasite-infected patients showed a significantly lower number of exacerbations, stable disability scores, and fewer new T2 and gadolinium enhancing (Gd+) lesions in brain magnetic resonance imaging (MRI). Consequently, new treatment paradigms based on the hygiene hypothesis were developed for autoimmune diseases, including the administering of non-pathogenic live microorganisms. The first clinical trials using *Trichuris suis* ova (TSO), a pig whipworm that is non-pathogenic in humans, in autoimmune diseases, such as inflammatory bowel disease, showed good tolerability and a striking suppressive effect on the autoimmune response [8]. Recently, Fleming *et al.* completed a phase I controlled trial that showed significant reduction of disease activity in RRMS patients under treatment with TSO [9]. We have also analyzed the effects of TSO in four patients with secondary progressive MS and observed a slight down-regulation of the Th1-associated cytokine pattern, especially interleukin (IL)-2, with a temporary increase of Th2-associated cytokines, such as IL-4. Furthermore, mild eosinophilia and changes in CD4+ and CD8+ T cells and natural killer (NK) CD56 bright cell numbers were evident [10].

A number of different hypotheses currently seek to explain how helminths influence autoimmune disorders. An increase in IL-10 and transforming growth factor-β and a decrease in IL-12 and interferon-γ-secreting cells have been shown in fresh peripheral blood mononuclear cells (PBMCs) of MS patients with a helminth infection [7]. The patients also had a higher number of CD4+CD25+FoxP3+ regulatory T cells [7] and IL-10-producing CD19+ B cells [11]. The B cells also produced higher levels of brain-derived neurotrophic factor and nerve growth factor. In the MS animal model experimental autoimmune encephalomyelitis (EAE), a reduced incidence and delayed onset of disease after infection with *Schistosoma mansoni* was seen, probably as a result of down-regulation of proinflammatory cytokines [12].

Overall, this research suggests that TSO is a promising therapeutic option in RRMS. We plan to investigate this further in a monocentric, prospective, randomized, placebo-controlled, double-blind phase II pilot study in patients with RRMS or clinically isolated syndrome (CIS) [13] using MRI and clinical assessment, and hypothesize that oral administration of 2,500 embryonated TSO every two weeks is superior to placebo administration.

## Methods/Design

### Trial design

TRIOMS is a monocentric, prospective, randomized, placebo-controlled, double-blind, phase II pilot study to be conducted at the Department of Neurology and the NeuroCure Clinical Research Center of the Charité - Universitätsmedizin Berlin. We plan to begin recruitment in Summer 2012. Fifty patients with RRMS or CIS, who have not undergone immunomodulatory treatment for at least 3 months, will be randomized to either TSO 2,500 ova or placebo to be taken every two weeks for 12 months as an immunomodulatory monotherapy. The detailed study design is based on the revised Consolidated Standards of Reporting Trials Statement [14,15].

The study is approved by the local ethics committee and the German competent authority (Federal Institute for Drugs and Medical Devices). The trial is registered at Clinicaltrials.gov (NCT01413243) and will be conducted in accordance with the Declaration of Helsinki in its currently applicable version, the guidelines of the International Conference on Harmonization of Good Clinical Practice (ICH-GCP), and the applicable German laws. All participants will be required to give written informed consent. The trial will be monitored according to ICH-GCP.

### Participants

The inclusion criteria for participation in the TRIOMS trial comprise the diagnosis of either definite RRMS according to the revised 2005 McDonald criteria [16] or CIS, an age of 18 to 65 years, a score of ≤4.0 on the Expanded Disability Status Scale (EDSS) [17], and disease activity on brain MRI (Table 1).

**Table 1:** Eligibility criteria

**Key inclusion criteria**	Diagnosis of RRMS in accordance to the revised McDonald criteria (2005) or CIS
	Aged 18 to 65 years, inclusive
	Expanded Disability Status Scale (EDSS) at screening score ≤4.0
	Ability to provide written informed consent
	Disease activity on brain defined by:
	(A) ≥1 gadolinium enhancing lesion on screening MRI or another MRI performed in the 12 months prior to screening OR
	(B) ≥1 new T2 lesion on screening MRI or on another MRI performed in the 12 months prior to screening in comparison to an earlier MRI performed in the previous 36 months OR
	(C) ≥1 enlarging T2 lesion on screening MRI or on another MRI performed in the 12 months before screening in comparison to an earlier MRI performed in the previous 36 months
	Adequate birth control by a contraception method with a PEARL-index <1 in women of childbearing potential
	Contraindication or intolerance for established standard immunomodulatory treatments with interferon-β or glatiramer acetate
	Information about established standard immunomodulatory treatments for MS by an independent neurologist and explicit decision of the patient against these treatment
	Stable neurological state at study inclusion without any signs of a relapse and without steriod therapy in the last 30 days
**Key exclusion criteria**	Any disease other than MS that may better explain the symptoms and signs
	Any other immunomodulatory or immunosuppressive treatment, for example, interferon-β, mitoxantrone, glatiramer acetate, natalizumab within the preceding three months
	Any significant uncontrolled disease, such as neoplasia, relevant liver disorder, active hepatitis B or C, and reduced liver function
	Relevant laboratory findings:
	• aspartate aminotransferase/alanine aminotransferase (ASAT/ALAT) >3 times of reference value
	• bilirubine >1.5 mg/dl
	• hemoglobin <8.5 g/dl
	• white blood count <2.5/nl
	• thrombocytes <125/nl
	• creatinine-clearance according to Cockroft-Gault-formula <110 ml/min (male) and <95 ml/min (female)
	Any severe medical conditions or additional autoimmune disease which requires a immunosuppressive or immunomodulatory treatment
	Any psychiatric or other condition that impedes the ability to provide informed consent or patient’s compliance
	Refusal of transmission of personal data
	Inability to complete an MRI scan
	Relapse within 30 days of trial entry
	Known allergies against components of TSO or placebo
	Participation in any interventional clinical trial in the last three months or during the participant in TRIOMS
	Pregnancy or breast feeding
	Concomitant medication with antihelminthic therapy
	History of small intestinal resection

The main exclusion criteria include any disease course other than RRMS or CIS, any disease other than MS that may better explain the symptoms and signs, any immunomodulatory or immunosuppressive treatment, such as interferon-β, mitoxantrone, glatiramer acetate, natalizumab, within the preceding three months. Additionally, the participants must not have any significant uncontrolled disease, such as neoplasia or cardiovascular, renal, hepatic, endocrine or gastrointestinal disease or any other significant disease that may preclude the patient from participating in the study (Table 1).

### Randomization and interventions

The eligibility of patients will be determined at the screening visit. At the baseline visit, patients who qualified for participation in the study will be stratified according to gender and disease course (RRMS/CIS) and randomized 1:1 into the two intervention groups. One group will receive TSO 2,500 eggs orally every 14 days, the other a placebo suspension orally every 14 days. Visits will be carried out after 3, 6, 9 and 12 months (Table 2). To ensure reliable monitoring of tolerability, telephone check-ups are planned after every dispensation if a patient does not visit the study center, because prior studies have shown frequent gastrointestinal adverse events or symptoms after therapy with TSO [18]. Optional follow-up visits will be offered at 15 and 18 months after randomization. If necessary, additional unscheduled visits can be performed at any time.

**Table 2:** Time schedule of regular study visits

	**Screening**	**Random (month 0)**	**Tel.V1 -6 (week 0, 2, 4, 6, 8, 10, 16, 20, 28, 32, 40, 44) ± 3 days**	**V1 (month 3) ± 10 days**	**V2 (month 6) ± 10 days**	**V3 (month 9) ± 10 days**	**V4 (month 12) ± 10 days**	**V5 (month 15) ± 10 days *(optional)***	**V6 (month 18) ± 10 days *(optional)***
Informed consent	x								
In/exclusion criteria	x	x							
Randomization		x							
Demographic data	x								
Pregnancy test	x								
Confirmation of MS diagnosis	x								
Relapse history	x	x		x	x	x	x	x	x
Medical history	x	x		x	x	x	x	x	x
Physical examination	x			x	x	x	x		x
Vital signs	x	x		x	x	x	x		x
Weight	x						x		x
MSFC	x						x		x
EDSS	x		x	x	x	x	x		x
Safety lab	x			x	x	x	x		x
Immunological sampling		x		x	x	x	x		x
MRI	x				x	x	x	optional	optional
AE/SAE				x	x	x	x	x	x
Questionnaires (fatigue, depression)	x	x			x	x	x	x	x
Drug supply		x	x	x	x	x			
Drug account					x		x		

### Outcome parameters

The primary endpoint is the cumulative number of new hyperintense lesions identified on T2-weighted brain MRI during the treatment period of 12 months. Additional secondary MRI endpoints comprise the volume of T2 hyperintense lesions and number and volume of contrast-enhancing T1 hypointense lesions, the proportion of patients without any new T1 hypointense and T2 hyperintense lesions, brain atrophy as determined by brain parenchymal fraction and normalized brain volume 18 [19], and changes in brain metabolism (NAA/Cr-quotient) as determined by MR spectroscopy [20,21]. All MRI investigations will be performed using a 3 Tesla MRI scanner (Siemens Healthcare, Erlangen, Germany. Other secondary clinical endpoints include the annualized relapse rate and the proportion of relapse-free patients. A relapse is defined as *de novo* development, aggravation or re-occurrence of a pre-existing neurological abnormality compatible with MS, which lasts a minimum of 24 hours, is separated by at least 30 days from a preceding clinical event and does not occur in the context of fever or infection. Further secondary endpoints are the disease progression as determined by EDSS (evaluated by an independent neurologist) [17], Multiple Sclerosis Functional Composite (MSFC) [22], depression as determined by the Beck Depression Inventory (BDI) [23] and fatigue as determined by the Fatigue Severity Scale (FSS) [24]. Assessment of all clinical and MRI endpoints will be performed by experienced evaluators blinded to both the clinical data and treatment allocation. Peripheral venous blood (serum, heparinized blood for flow cytometry and PBMC isolation) will be sampled prior to and during the intervention to assess the effect on cellular and soluble components of the immune system. In detail, we plan to monitor the Th1/Th2/Th17 balance by concanavalin A stimulation of fresh heparinized blood and determination of cytokines using the cytokine bead array. In fresh heparinized blood, we will measure the frequency of B cells, CD4+ and CD8+ T cell subpopulations, innate immune cells and quantify the activation and homing markers on these cells by multicolor flow cytometry. We will also assess the number of IL-10-producing B cells by stimulation of fresh isolated PBMCs with 0.1 mg/ml CpG ODN2006 for 72 hr and will add phorbol myristate acetate and ionomycin (PMA+iono) for the last 6 hr and brefeldin A for the last 4 hr in order to block cytokine excretion. Detection of IL-10 producing cells is planned as intracellular staining of IL-10 on CD 19 expressing cells by multicolor flow cytometry. Alternatively, IL-10 we will be measured in the culture supernatants by ELISA with aliquots of supernatant taken from cell cultures before the addition of brefeldin A and PMA+iono to the cultures.

### Sample size

Because the present data on the effect of TSO on the development of new T2 hyperintense lesions on brain MRI, which is the primary endpoint of the TRIOMS study, are insufficient for an exact statistical sample size calculation, we have designed the investigation as a pilot study with an *a priori* determined sample size of 25 patients per intervention arm, giving a total of 50 patients. The results of this exploratory analysis could serve as the basis for a further prospective randomized study with a statistically justified sample size.

### Blinding

Both the patients and all study personnel will remain blinded throughout the complete treatment period of 12 months. Treatment allocations will only be disclosed after the final database lock or in case of emergency. The active study therapy and placebo bottles will be supplied by OVAMED GmbH and labeling of bottles and randomization of patients will be performed in the central pharmacy of the Charité - Universitätsmedizin Berlin. The patients will mainly be attended to by a “treating physician”, responsible for evaluating the inclusion or exclusion criteria, adverse events, relapses, side effects and so on. All neurological examinations will be performed by an independent “evaluating physician”. To separate the physicians’ function in a threating and an evaluating position in order to prevent unblinding is necessary because patients under therapy with TSO commonly report gastrointestinal side effects that may point the physician to the verum therapy. Similarly, evaluation of all other paraclinical parameters, including MRI, will be conducted by independent examiners. Any patient-related crosstalk between treating and evaluating physicians or examiners will be prohibited unless required for safety reasons. A simple unblinding procedure has been designed, which allows for rapid unblinding of a patient in case of a medical necessity. If the patient is unblinded, he or she will be automatically excluded from the study.

### Statistical methods

Endpoints will be evaluated by both intention-to-treat- and per-protocol-analyses. Statistical tests and presentation will be appropriate to the scaling and distribution of the respective variables. Because TRIOMS is planned as a pilot study, there is a statistical analysis of endpoints, but *P*-values will be considered exploratively without confirming generalization of hypotheses about the study treatments. Due to the restricted sample size of the pilot study, sufficiently powerful statistical analysis is not possible. The primary end point will be analyzed using Fisher’s exact test. Secondary endpoints will be analyzed non-parametrically using Mann–Whitney-U-test, due to the small sample size and possible deviations from normality. Frequencies will be compared with the Fisher’s exact test.Because of the exploratory character of the analysis, alpha-adjusting will not be performed. The test level for statistical significance of differences between both treatment arms is defined as *P* = 0.05 (two-sided) for all tests. For statistical analyses use of the following software is planned: SAS, version 9.4 (SAS Institute Inc. Cary, North Carolina, US); SPSS, version 20 (IBM, Armonk, New York, US) and StatXact 6 from CYTEL (Cytel Inc., Cambridge, Massachusetts, US).

## Discussion

Approved medications for MS, such as beta interferons or glatiramer acetate have limited therapeutic efficacy or, in the case of natalizumab, mitoxantrone or fingolimod, may be associated with severe side effects. The ideal drug for patients with MS should be highly effective with few and clinically insignificant side effects and should be administered orally. Existing epidemiological and preclinical data suggest that helminth infections are protective in autoimmune diseases, such as MS, and the first clinical trials with oral administration of pig whipworm *Trichuris suis* eggs in MS revealed good tolerance and surprising clinical effects [9,10]. Mechanisms of how helminth infections provide protection in autoimmune diseases are manifold and comprise immunosuppression attributed to an increased activity of regulatory T cells (Treg cells), alternatively activated macrophages and, more recently, IL-10 producing regulatory B cells. Further, helminth infections induce the production of Th2 related cytokines, such as IL-4, IL-5, IL-9 and IL-13, with anti-inflammatory qualities [25]. In addition, competitive mechanisms for antigen processing, antigen binding and for essential cytokines may exist between anti-infectious and autoimmune responses [26].

Here, we present a study design that has the potential to substantially contribute to the evaluation of the efficacy and tolerance of TSO in MS patients. The randomized, controlled, double-blind study design and the implementation of independent evaluation of outcome parameters fulfill the current criteria for a high quality clinical phase II trial in MS [14,15]. A limited sample size was chosen to reduce the number of patients on placebo and on a possibly ineffective therapy. On the other hand, this limited sample size may not be sufficient to show a statistically significant superiority of TSO compared with placebo. The placebo design was chosen to evaluate the sole effect of TSO on MS activity and to minimize the risk of uncontrolled immunosuppression that could be relevant in a treatment with TSO and other immunosuppressive or immunomodulatory therapies. Fleming *et al.* showed a reduction of Gd+ lesions in MRI under therapy with TSO in MS patients after three months of therapy and an increase of lesions in the post-treatment period, suggesting that a permanent therapy is desirable for treatment of a chronic disease like MS [9]. Therefore, we have chosen a relatively long therapy duration of 12 months to confirm on the one hand the clinical effects of TSO on MS activity and to show on the other hand that this medication fulfills safety aspects for long time use.

In conclusion, TSO has the potential as an effective, safe and orally available treatment option in MS and the TRIOMS trial may help to establish the first probiotic therapy in MS that is based on the hygiene hypothesis.

## Trial status

Start of recruitment September 2012.

## Abbreviations

AE: Adverse event; ALAT: Alanine aminotransferase; ASAT: Aspartate aminotransferase; BDI: Beck Depression Inventory; CIS: Clinically isolated syndrome; CNS: Central nervous system; EAE: Experimental autoimmune encephalomyelitis; EDSS: Expanded disability status scale; FSS: Fatigue Severity Scale; ICH-GCP: International Conference on Harmonization of Good Clinical Practice; IL: Interleukin; MS: Multiple sclerosis; MSFC: Multiple sclerosis functional composite; MRI: Magnetic resonance imaging; NK: Natural killer; PBMCs: Peripheral blood mononuclear cells; RR: Relapsing-remitting; SAE: Serious adverse event; TRIOMS: *Trichuris suis* ova in relapsing-remitting multiple sclerosis and clinically isolated syndrome; TSO: *Trichuris suis* ova; V: Visit.

## Competing interests

BR participated in meetings sponsored by and received lecture honoraria from Biogen Idec, Bayer, Merck Sorono and Teva Pharmaceuticals, and receives research support from Bayer Schering Pharma. FP received travel support and speaker honoraria by Bayer Healthcare, Novartis, Teva, Biogen Idec, Sanofi Aventis and Merck Serono. JD received travel support, speaker honoraria and research support by Novartis and Bayer Vital. The authors declare that they have no competing interests.

## Authors’ contributions

BR designed the study, drafted the study protocol and drafted the manuscript. SO was in charge of all regulatory affairs and critically revised the study protocol and manuscript. KDW is the biometrician of the study and critically revised the study protocol and manuscript. FP designed the study and also critically revised the study protocol and manuscript. JD critically revised the study protocol and manuscript. All authors have given final approval of the version to be published.
